# Ecological drivers of breeding periodicity in four forest neotropical eagles

**DOI:** 10.1038/s41598-023-31274-8

**Published:** 2023-03-16

**Authors:** Marcel Henrique Blank, Paulo Roberto Guimarães Jr, Lucas Ferreira do Nascimento, Ricardo Jose Garcia Pereira

**Affiliations:** 1 Grupo de Estudos para Multiplicação de Aves, Departamento de Reprodução Animal, Faculdade de Medicina Veterinária e Zootecnia, Universidade de São Paulo, Av. Duque de Caxias Norte, 255, Pirassununga, SP 13635-900 Brazil; 2 Departamento de Ecologia, Instituto de Biociências, Universidade de São Paulo, Rua Do Matão 321, Travessa 14, São Paulo, SP 05508-090 Brazil; 3 Programa de Pós-Graduação em Ecologia, Instituto de Biociências, Universidade de São Paulo, Rua do Matão 321, Travessa 14, São Paulo, SP 05508-090 Brazil

**Keywords:** Reproductive biology, Animal physiology

## Abstract

We explore the potential factors that affect clutch initiation in four Neotropical large raptors (Harpy eagle—HE, Crested eagle—CE, Ornate hawk-eagle—OHE, and Black hawk-eagle—BHE) by analyzing 414 clutch events mostly obtained from captive individuals. Differences in how clutch initiation is associated with changes in photoperiod were found between HE and both hawk-eagles, and between CE and BHE. Changes in temperature at the time of clutch initiation only differed between HE and OHE, whereas changes in precipitation varied between BHE and all other species. Principal Component Analysis of these environmental cues showed that ellipses in the dataset of each species overlap, but only ellipses from CE and OHE had the same variation trends. This means that although these species live under similar ecological conditions, they exhibit three different patterns of response to environmental cues. Apparently, these patterns are not associated with phylogenetic relatedness because species belonging to the same clade do not show the same response pattern. Diet diversity analysis revealed that HE has the least varied diet, and CE and OHE the most varied diet. The fact that species who fit the same reproductive timing response to environmental cues show similar diets leads us to hypothesize that breeding in these eagles was most likely shaped by food availability.

## Introduction

Breeding is one of the most energy costly activities in a bird’s annual cycle. Therefore, timing of reproduction occurs in a period when food is abundant, favoring offspring production and survival^1,2^. In temperate regions, selection favoring breeding times associated to resource availability leads to bird species exhibiting short and well-defined breeding seasons during increasing daylengths, whereas their tropical congeners tend to have longer breeding seasons due to lower variability in climate and food resources^3–6^. In parallel, birds in the tropics show greater variability among species, and even individuals, in their reproductive timing compared to mid- and high-latitude birds^6^. These patterns indicate that birds living in lower latitudes and environments with high year-to-year variability in seasonality tend to incorporate short-term cues (temperature, food, nest site availability, etc.) in a higher degree into their reproductive regulation^3,4,7^. Such feature confers a plasticity to the photoperiodic template that enables these species to breed as long as environmental conditions are favorable, even under short photoperiods^8,9^.

However, despite the higher species diversity at lower latitudes, almost all knowledge on seasonal regulation of reproduction in tropical birds derives from passerines^1,4,7,8,10–15^, leading to a phylogenetic bias that disregards other avian orders. One group that illustrates well this gap is Acciptridae (hawks, eagles, kites, harriers, etc.), a family comprising 69 species in the Neotropics whose sizes vary greatly and prey items range from insects to medium-sized mammals^16^. When compared to Passeriformes, many diurnal raptors display a great deal of variation in their ecological, social and life-history variables (e.g., most are solitary, resident, monogamous, and prey on other vertebrates), which possibly affected the evolution of their endocrine control mechanisms for reproduction. In addition to these multiple factors affecting endocrine control mechanisms, some aerial predators are long-lived animals (over 20 years) and it is likely they would invest less into each reproductive event than short-lived species, which often show a shorter number of reproductive events. Thus, it is expected for long-lived birds to show intermittent breeding^17–21^. Finally, large raptors such as Neotropical eagles exhibit longer rearing periods with juveniles staying near to their nests for up to 12 months^22^, circumstances that possibly contribute to intermittent breeding, by uncoupling breeding periods from within-year seasonality. Unfortunately, as far as we know no studies on breeding biology of Neotropical accipitrids have focused on how environmental cues modulate their reproductive decisions.

For all the above reasons, our aim is to understand what factors modulate the reproductive decisions in four large Neotropical accipitrids. For this, we gathered data on timing of clutch initiation and nestlings from Harpy eagles (*Harpia harpyja*—HE), Crested eagles (*Morphnus guianensis*—CE), Ornate hawk-eagles (*Spizaetus ornatus*—OHE) and Black hawk-eagles (*Spizaetus tyrannus*—BHE), in an attempt to: (1) characterize their breeding seasonality; (2) determine whether or not reproductive patterns are shared among related species; and (3) examine their reproductive response to different environmental stimuli (photoperiod, temperature, precipitation). Additionally, because diet can be important to the time of breeding, we investigated how diet varies through these four eagle species and if it is associated with breeding timing. By analyzing the breeding activities of these raptorial species, we expect to enhance our understanding on how the life-history strategies of Neotropical eagles have evolved to extrinsic constraints.

## Results

**Figure 1:**
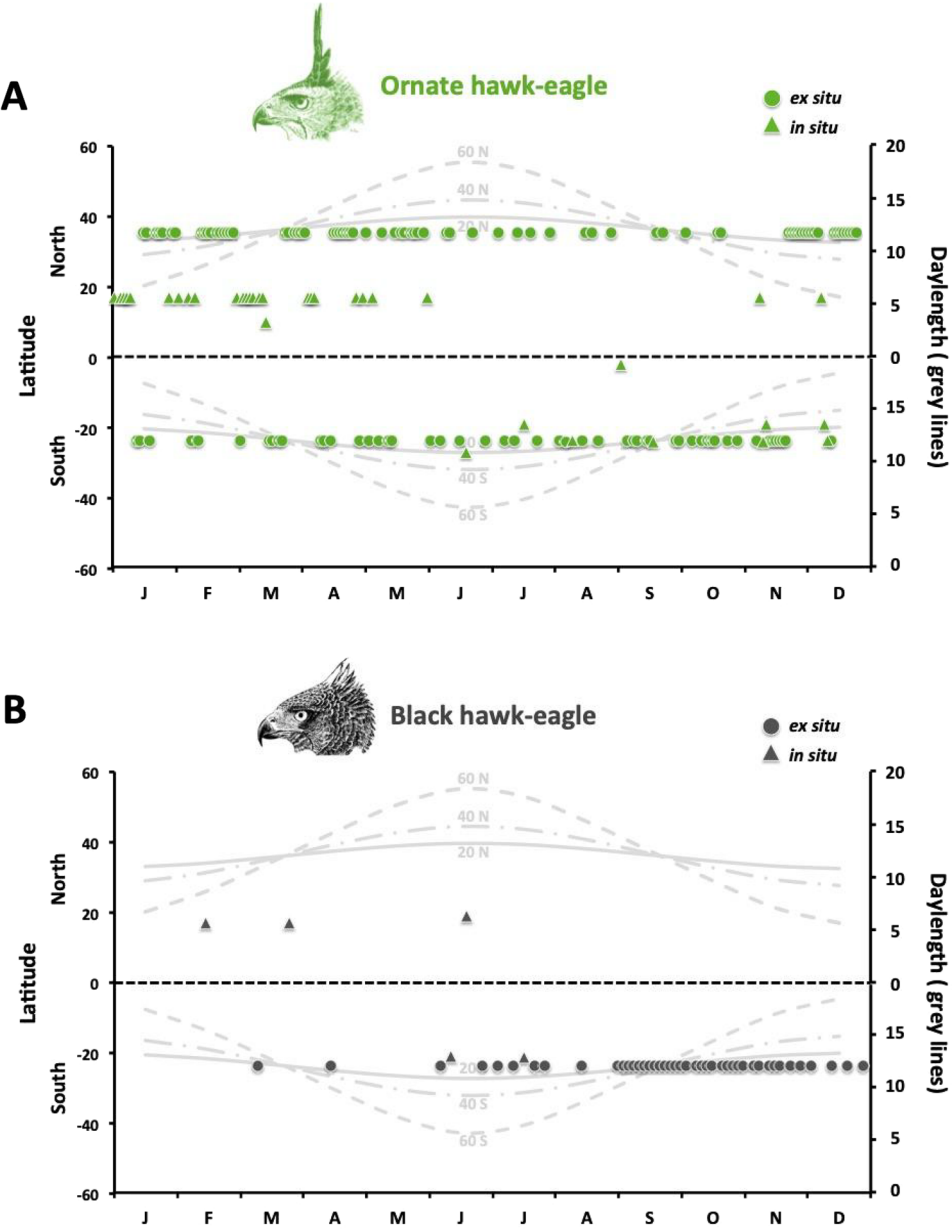
Annual distribution of clutches in (**A**) Ornate hawk-eagle (*Spizaetus ornatus*) and (**B**) Black hawk-eagle (*Spizaetus tyrannus*) females. Circles and triangles represent clutch events recorded in captivity and in the wild, respectively. Solid, dash-dotted and dashed gray lines depict annual variations in daylength at latitudes 20°, 40° and 60° of the northern and southern hemispheres.

**Figure 2:**
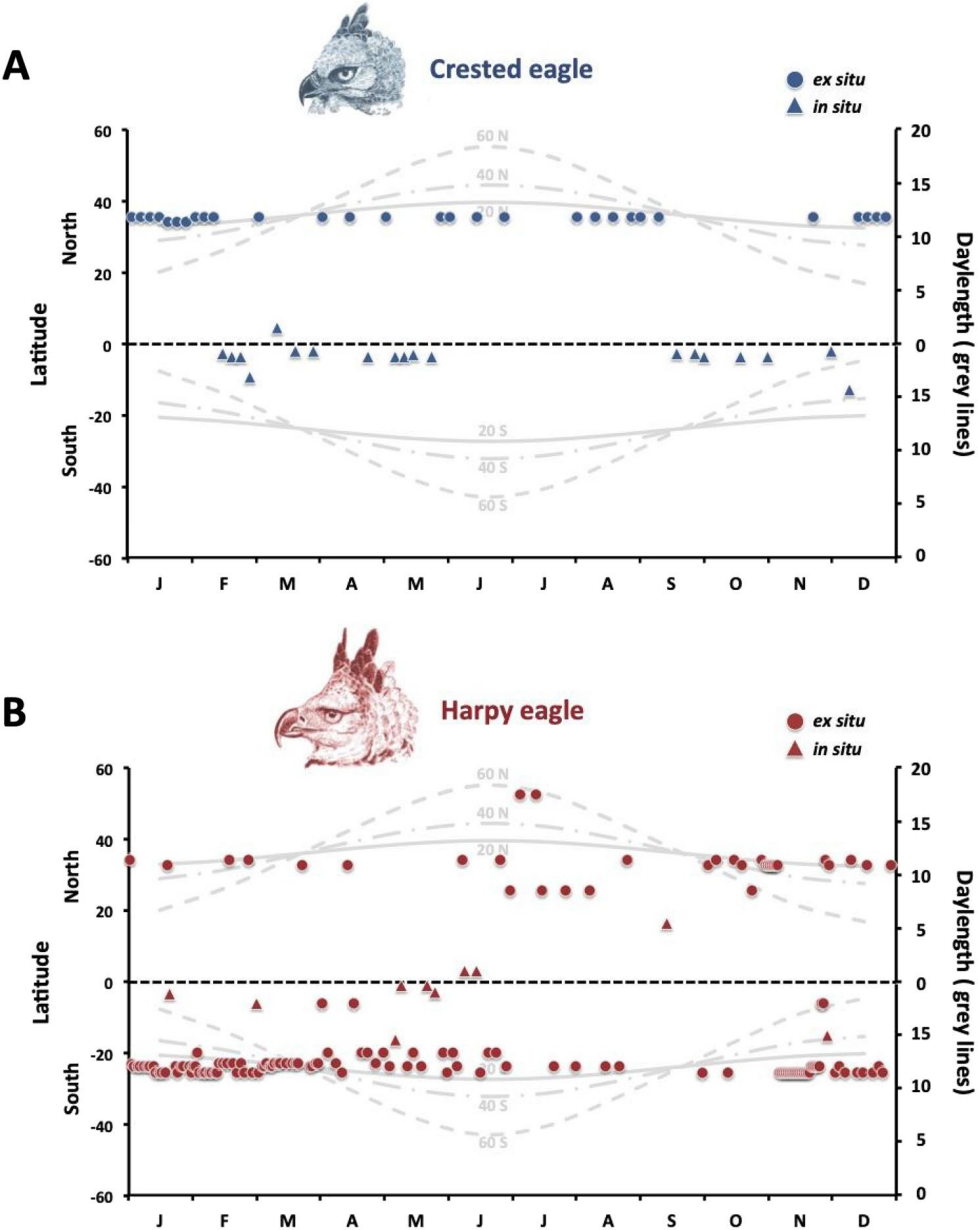
Annual distribution of clutches in (**A**) Crested eagle (*Morphnus guianensis*) and (**B**) Harpy eagle (*Harpia harpyja*) females. Circles and triangles represent clutch events recorded in captivity and in the wild, respectively. Solid, dash-dotted and dashed gray lines depict annual variations in daylength at latitudes 20°, 40° and 60° of the northern and southern hemispheres.

**Figure 3:**
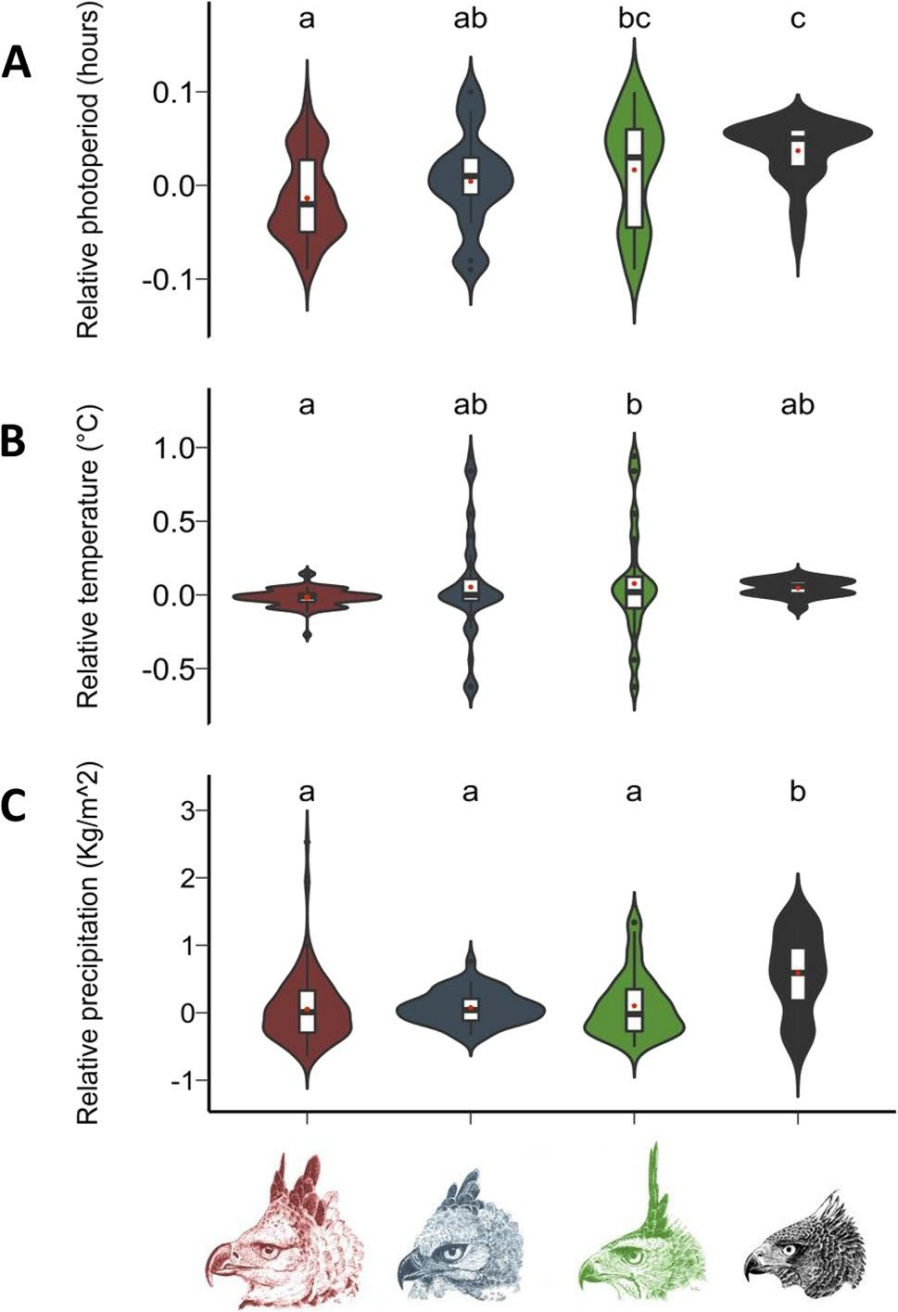
Violin plots of the relative changes in (**A**) photoperiod, (**B**) temperature and (**C**) precipitation in which clutch initiation occurred in the four Neotropical species surveyed in this study. Red, blue, green and black violin plots represent values obtained from Harpy eagles, Crested eagles, Ornate hawk-eagles and Black hawk-eagles, respectively. Black horizontal lines indicate medians whereas red dots depict means. Boxes illustrate interquartile range and outliers 1.5 times the interquartile range from the box are shown as dots. Different letters indicate statistical differences between species within the same environmental factors (*P* < 0.05).

Our data collection managed to identify 414 clutch initiations of which 156, 48, 159 and 51 were from HE, CE, OHE and BHE, respectively (where 93.5%, 60.4%, 77.3% e 84.3% were from captivity, respectively) (Supplementary Fig. 1, Supplementary Tables S1 and S2). Our survey also showed that all captive individuals were kept under ambient light and temperature conditions, and were fed a diet consisting basically of rabbits and rats, and on rare occasions chicken and quail for OHE and BHE (8–10% body weight, 3–5 times a week). Furthermore, all individuals sampled here had been in captivity for more than 5 years. Regardless of latitude or origin (ex situ or in situ) where individuals inhabited, clutch initiation in all four species was observed on both increasing and decreasing daylengths (Figs. 1 and 2). However, the extent to which these events were distributed along the year varied greatly among species. Such interspecific differences were later supported statistically considering the relative changes in photoperiod, temperature and precipitation. Clutches in HE occurred mainly during decreasing daylengths, differing from OHE and BHE (but not from CE) in terms of relative changes in photoperiod (Fig. 3A, Supplementary Table S3). Furthermore, relative changes in the photoperiod in which clutch initiation occurred also differed significantly between CE and BHE (Fig. 3A). Regarding clutches and relative changes in temperature, we found differences only between HE and OHE, with events in HE tending to happen during decreasing temperatures and in OHE during increasing temperatures. (Fig. 3B, Supplementary Table S3). Interestingly, BHE set itself apart from the others with respect to clutches and relative changes in precipitation, exhibiting a tendency to lay more during increased rainfall (Fig. 3C, Supplementary Table S3). Further analyses using only the captive data were quite similar, with the exception of clutch initiation in relation to relative photoperiod which was no longer different between CE and BHE (Supplementary Fig. 2, Supplementary Table S4). Clutch initiation data from captive and free-ranging individuals within the same species only varied in OHE and BHE for relative changes in photoperiod and precipitation, respectively (Supplementary Fig. 3).

**Figure 4:**
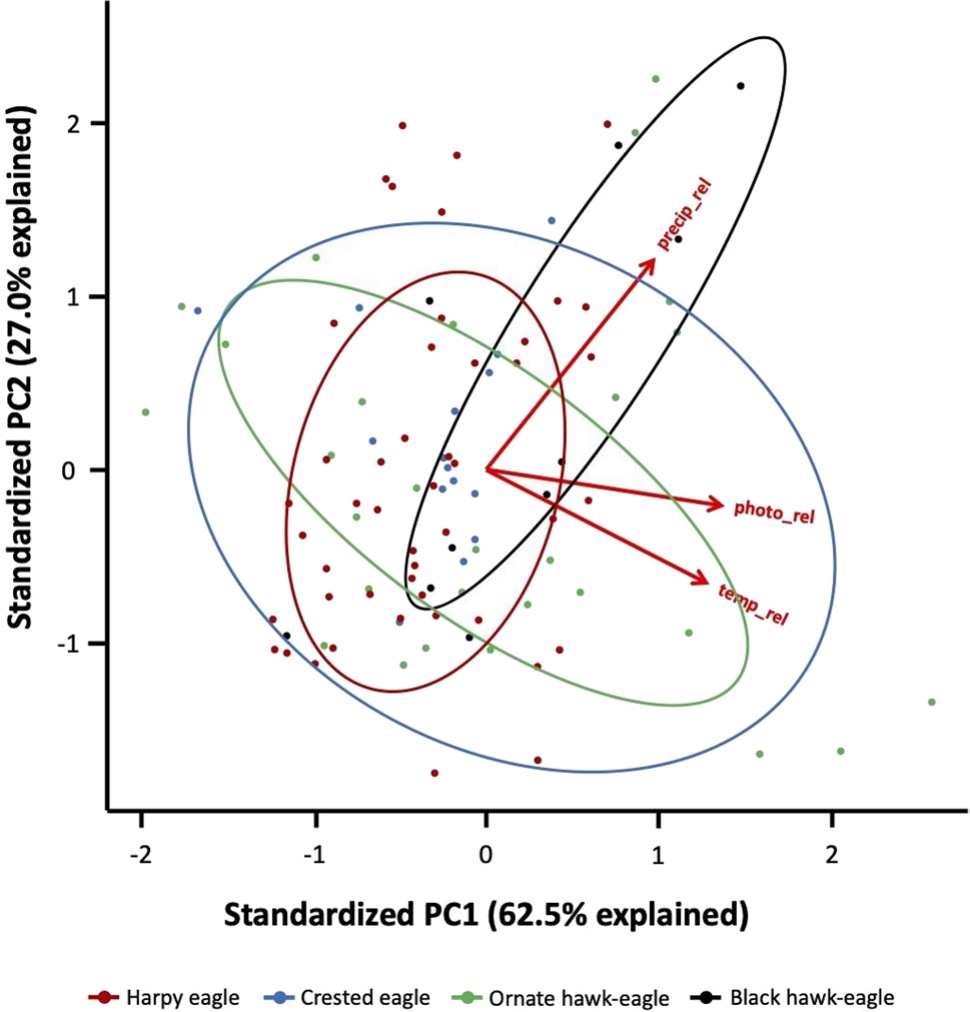
Principal Component Analysis (PCA) of the variables relative changes in photoperiod, temperature, and precipitation. The first principal component (x axes) explained 62.5% of the data variation and the second principal component explained 27% of the variation of the data variation. The ellipses define the region that contains 68% of the data for each eagle. The red arrows represent the loadings of each variable (relative photoperiod, relative temperature and relative precipitation). The values of the data in terms of the principal components were multiplied by -1 to facilitate the visualization.

Next, based on the Principal Component Analysis (PCA), we observed that PC1, that was associated with all environmental factors, was able to explain 62.5% of the variation in clutch initiation distribution while PC2, mainly associated with relative precipitation, accounted for 27.0% (Fig. 4., Supplementary Table S5). Another finding given by the PCA refers to the overlapping of the ellipses obtained from the data of each species that, despite being detected, also suggest three different patterns of response to the analyzed factors. The only ellipses that showed the same variation trend were those from CE and OHE (Fig. 4). PCA analysis using only captive data have not qualitatively changed our results (Supplementary Fig. 4, Supplementary Table S6).

**Figure 5:**
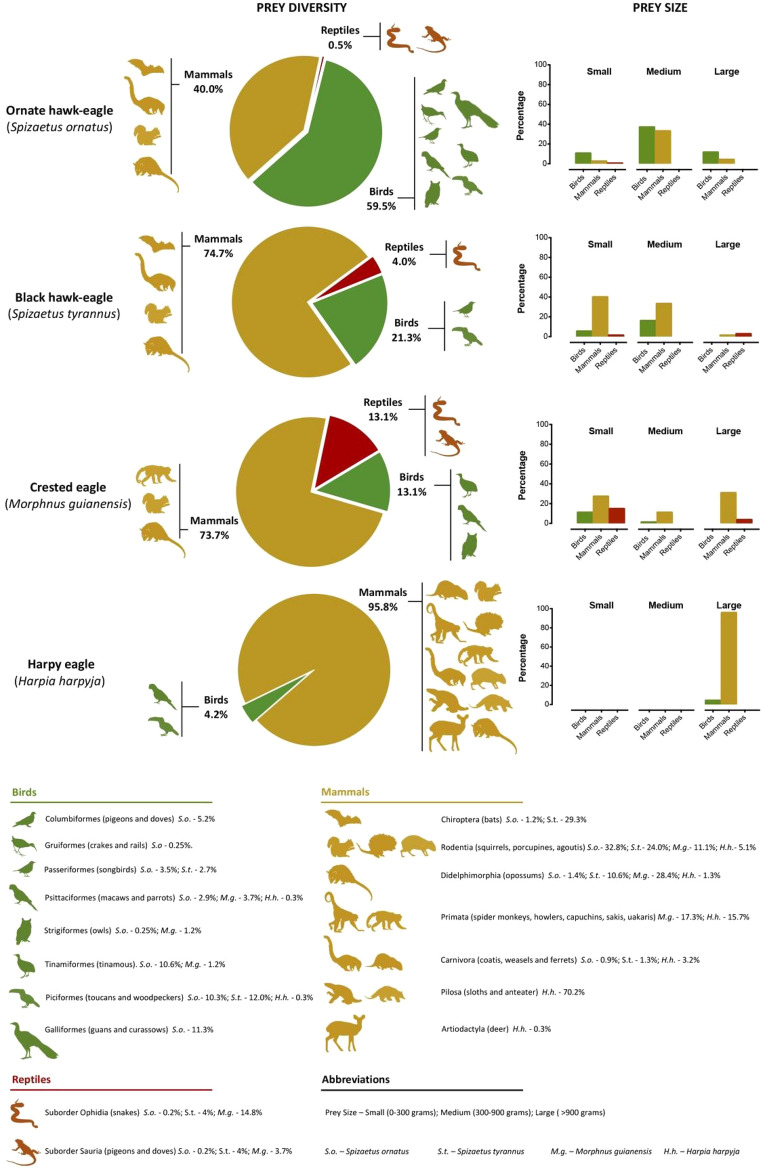
Prey type and size of the Ornate hawk-eagle, Black hawk-eagle, Crested eagle and Harpy eagles. The pie charts illustrate the percentages of mammals, reptiles and birds consumed by each species and the silhouettes represent the orders of the animals they prey on. The bar charts show the percentages of prey by size category (Small—0–300 g; Medium—300–900 g; and Large—> 900 g). In the lower panel, the percentages next to animal silhouettes detail how much a particular order accounts for the total prey consumed by each raptor. S.o.—Ornate hawk-eagle (*Spizaetus ornatus*); S.t.—Black hawk-eagle (*Spizaetus tyrannus*); M.g.—Crested eagle (*Morphnus guianensis*); and H.h.—Harpy eagle (*Harpia harpyja*).

In an attempt to understand whether or not these patterns were somehow linked to the evolutionary history of these eagles, we estimated the phylogenetic distance among these four eagles. They shared a common ancestor 32.659 million years ago, but HE and CE are more closely related in evolutionary time than OHE and BHE, indicating that response patterns are not shared by species belonging to the same clade (Supplementary Fig. 5, Supplementary Table S7). Finally, our dietary survey revealed that although these birds are top predators and have similar distributions in the Neotropics, they display different prey diversity (Fig. 5, Table 1). The Shannon Diversity Index was found to be low for HE, medium for BHE, and high for CE and OHE.

**Table 1:** Diet diversity of the four Neotropical eagles studied.

Species	Ornate hawk-eagle (*Spizaetus ornatus*)	Black hawk-eagle (*Spizaetus tyrannus*)	Crested Eagle (*Morphnus guianensis*)	Harpy eagle (*Harpia harpyja*)
Number of items				
Class	0.701	0.504	0.826	0.173
Order	1.855	1.366	1.572	0.910
Biomass				
Class	0.687	0.391	0.894	0.186
Order	1.994	1.091	1.750	1.730

## Discussion

Seasonality and other periodic environmental changes have shaped a variety of life-history strategies in birds so that energetically demanding processes do not overlap, and breeding occurs when food resources are more readily available^23^. Therefore, most birds use predictable external cues to time these physiological processes, and generally variations in photoperiod serve to synchronize circannual rhythm of life-history changes (Dawson, 2008a). However, the degree to which these external cues are incorporated into the control of the annual cycle in birds depends on latitude and habitat predictability, given their relationship with food abundance^9,15,24^. Here, we examined how breeding in four large forest raptors is affected by extrinsic factors such as photoperiod, temperature and precipitation. Our survey revealed long breeding seasons for all species irrespective of latitude or origin (captivity or wild) with egg records on both increasing and decreasing daylengths, even in individuals kept longer than 7 years at high latitudes. Despite being the only raptor without data from temperate regions, only BHE showed a clear clutch concentration during increasing daylengths. Apart from that, comparisons among species with reference to clutch dates showed no common trend of response to any of the environmental stimuli analyzed, suggesting two possibilities: (1) the evolution of species-specific responses to relative changes in photoperiod, temperature and precipitation or (2) a more prominent role of nonphotoperiodic cues (possibly prey abundance) in the control of their reproductive cycles.

If the development of different response mechanisms had happened among these species, one would expect that evolutionary closer raptors would at least exhibit similar reproductive patterns or that their respective clutch dates would be more linked to a particular environmental cue. However, we notice that instead of those belonging to the same clade displaying a similar response pattern, the opposite occurred, with species from different clades showing the same trend (e.g. CE and OHE). In contrast, food availability is congruent with patterns of breeding timing. Thus, it appears more likely that food availability acts as a potent stimulator/ inhibitor for reproductive activity in these predators, since PCA analysis revealed three trends of response that do not seem to be explained in evolutionary terms.

A large body of work on regulation of breeding in birds argues that species living at lower latitudes tend to rely more on nonphotoperiodic information to adjust their reproductive decisions than those from higher latitudes^3,4,7,25,26^. Photorefractoriness plays an important role not only in the length of breeding seasons but also in their asymmetry relative to the photoperiod^3^. Overall, it is believed that birds with longer or less predictable breeding seasons become relatively photorefractory, and birds that rely on unpredictable food resources are opportunistic breeders^1,3,27^. Applying that reasoning, some researchers suggest that several tropical species remain in a physiological state of "readiness to breed" for much of the year, using nonphotoperiodic factors such as food abundance to time their breeding^9^. In opportunistic breeders, it has been found that food can even override photoperiod in the regulation of testicular growth^25^.

The food availability hypothesis is supported by the fact that captive individuals from all species studied here laid more evenly throughout the year than free-living ones, indicating that the lack of variation in food supply triggers a stimulatory effect on these birds, even at higher latitudes. A former study detected peaks of fecal androgens, progestogens and estrogens in pairs of HE in almost all seasons of the year, leading authors to the conclusion that this species was not absolutely photorefractory (25^o^S latitude)^28^. Unfortunately, so far no research has assessed in detail the reproductive activity or endocrinology of the other three Neotropical eagles, due to the difficulties in finding a reasonable number of breeding pairs kept under similar management and environmental conditions. The idea of food availability acting as a proximate cue in adjusting the timing of breeding has been previously discussed by Hahn et al.^29^, who distinguished among several features of food availability (p.e. amount, type, quality, and predictability). According to these authors, there are several examples coming from a variety of field and laboratory studies that show the proximate effects of food quality or specific food types on the reproductive axis^14,30–33^. More specifically in raptors, it has been previously reported that free-living Common kestrels (*Falco tinnunculus*) advanced laying when extra food was given, whereas female Ospreys (*Pandion haliaetus*) delayed laying in 20 days by receiving 32% less fish per day from their mates^34,35^.

Differences in food availability may also explain why clutch initiation in captive and free-living populations of OHE and BHE varied differently in relation to relative changes in photoperiod and precipitation, respectively. However, it is curious to note that the same differences between captive and wild were not observed for HE and CE, possibly because laying in these species was much more spread out over the year regardless of the origin of the birds. Of course, we cannot fail to consider the disparities in the data of at least 3 of the 4 species analyzed with the overwhelming majority coming from captivity for HE, OHE and BHE, a situation that probably led to latitudinal, nutritional and stress effects. Additionally, it is interesting to observe that the vast majority of captivity data came from subtropical and temperate latitudes, and yet there was no common pattern of clutch initiation for all species with respect to photoperiod, temperature or precipitation. Another indication that food may be a powerful stimulus in their reproductive decisions relies on the fact that species who showed close response patterns to environmental cues (OHE and CE in the PCA analysis) had the highest values in the Shannon index. On the other hand, the least diverse and intermediate species in terms of diet (HE and BHE, respectively) demonstrated different response patterns not only among themselves but also in relation to the other two species. Thus, it seems plausible to assume that eagles relying on a wider range of prey to feed their young have broader opportunity windows for breeding than those dependent on a narrower number of prey species.

A further relevant point that most likely interferes with breeding periodicity and timing in these raptors refers to the duration of the rearing period. The literature on these birds describes that juveniles remain in the vicinity of the nest (within 100 m) for up to 12 months, and successfully nesting pairs can breed during every second or third year^22,36,37^. Long rearing periods and intermittent reproduction are traits shared by other large and long-lived birds such as albatrosses and king penguins, and are seemingly linked to body mass and recovery of body condition^21,38^.

In albatrosses, biennial breeding was associated in part to their large size because it leads to longer rearing periods, but also to their distant foraging trips given their impact on body condition of parents after rearing^21^. Likewise, all eagles studied here experience high energetic demands during the chick-rearing phase, performing several hunting forays into the forest that may considerably lower the body condition of the parents. This extra cost in terms of energy forces individuals to recover body condition in order to breed again, which brings us back to food availability acting as an important proximate regulator of reproductive physiology in these birds of prey. The mechanisms underlying this connection between body condition and reproductive axis are still being unraveled in birds, and so far we know that metabolic state modulates timing of breeding both through the effects of glucorticoids, leptin and ghrelin both on the release of gonadotropin inhibiting hormone (GnIH) and through their direct effects on the gonads^24^.

In summary, our data show that HE, CE, OHE and BHE have long breeding seasons regardless of latitude or origin (captivity or wild) and without a common pattern of response to relative changes in photoperiod, temperature and precipitation. The only patterns that resembled each other were of species that do not belong to the same clade but show high diet diversity. It appears that food in these raptors plays a dual role in the physiological regulation of reproduction, acting as both an ultimate and proximate factor of the reproductive timing. Despite the logistical and sampling difficulties of conducting experiments in captivity or monitoring in the wild, further research addressing photoperiod or food manipulation is needed to advance our understanding of the mechanisms controlling reproductive timing in these Neotropical eagles.

## Material and methods

To produce a dataset from captive individuals, we sent reproductive questionnaires to over 25 institutions worldwide that, according to our survey, had historical records of breeding activity (eggs and nestlings) of the studied species. These questionnaires consisted of a set of questions covering data on the animals (origin – captive-bred or wild caught, age, time in captivity), enclosures (size, nests, shaded areas, and ambient or controlled conditions), diet (type, amount and frequency of feeding) and reproduction (pairing, copulation, egg laying and chick rearing). Additional information about in situ and ex situ reproduction of the four Neotropical eagles was achieved by performing searches in four online databases of peer-reviewed publications (Web of Science, Google Scholar, JSTOR and PubMed) using common or scientific names of these species as well as the terms “Neotropical raptors” or “Neotropical birds of prey”, in combination with the following keywords: ‘breeding’, ‘reproduction’, ‘reproductive’, ‘nest’, nestling’, and ‘fledgling’^39–62^. Furthermore, we used image records of nestlings published in the WikiAves website (www.wikiaves.com.br), which includes dates and locations of each photograph. Then, dates of egg-laying were determined by subtracting egg incubation time and nestling age, which were defined through photographic guides from eagles of the same species hatched in captivity. In order to avoid eggs belonging to the same clutch being counted as individual egg-laying events, we only considered eggs that were laid at least 8 days apart from each other (in both captive and free-ranging records).

Climatic data were obtained from National Centers for Environmental Prediction (NCEP) in which we used a climate forecast system reanalysis supplied for^63^. Briefly, between 1979 and 2010, monthly means of temperature and rainfall were analyzed according to the following standards. For temperature, we use the surface layer temperature with a spatial and temporal resolution of 2.5° × 2.5° to each 6 h respectively. Regarding rainfall, we used precipitation accumulated to each 1 h for generating a total daily precipitation. The spatial resolution used was 0.5° × 0.5° and for both variables, the employed geographical limits were 73° north to 57° south × 176° west to 174° east. Daylength (DL) was calculated for each latitude using the following formula: DL (hours) = 2/15*arccos (-tangent (L)*tangent(δ)), where, L is the latitude of the location, and δ is the declination of the location on the day of interest. The declination, in turn, was calculated according to the formula: δ = 23.45*sen(360/365*(284 + n), where n is the number of the day of interest in the year.

Data related to the diet of these raptors was gathered through literature review (using the keywords: ‘diet’, ‘dietary’, ‘prey ‘, ‘food’, ‘feeding habits’, ‘feeding strategy’ and ‘feeding behavior’), mainly from articles in which food items were directly observed or collected in or around nests^44–46,49,51,55,64,65^. Prey biomass was estimated based on the same articles, but if such data were not available in this material we reviewed information provided by^22,36,66,67^.

All analyses were performed using R Statistical Software^68^. In order to verify whether or not clutch initiation in these species occurred preferentially in the increase or decrease of a given environmental factor, we chose to analyze relative changes in photoperiod, temperature and precipitation rather than the absolute values in which laying events took place. The relative changes for these factors were calculated by the following formula: V_rel_ = (V − V_bef_) / V_bef_, where V_rel_ is relative value of the variable (photoperiod, temperature or precipitation), V is the value of variable in the date of clutch initiation, and V_bef_ is the value of variable one month prior to the clutch initiation. The residuals of the linear models between the species and the relative values for these environmental variables are homoscedastic and, therefore, these variables were not transformed. Analysis of variance (ANOVA) was used to assess the significance of statistical differences among species regarding relative changes in photoperiod, temperature and precipitation, and Tukey post-hoc tests were performed for multiple comparisons between each pair of species. These tests were subsequently run using only the captive data. Besides, we performed Student's T-test to determine if there were differences in the relative changes in photoperiod, temperature, and precipitation between captive and free-living individuals within the same species. Then, because all environmental factors are likely to be correlated, we performed a Principal Component Analysis (PCA) of the relative changes in photoperiod, temperature, and precipitation. This analysis allowed us to see the overall patterns of covariation of our data, identifying which species show similar patterns of covariation in the environmental factors associated with breeding timing t and which are very different. In order to investigate the phylogenetic relationship between the species, we calculate the phylogenetic distance (in millions of years) between the species using the *distTips* from *adephylo* R package^69^. Calculations of the phylogenetic distances among species were based on phylogenies estimated by^70^ which are available in Birdtree.org. Lastly, we estimated the Shannon diversity index in relation to the diet for each species in order to investigate how diets vary among these raptors. This index is calculated using the following equation: H = − ∑^s^_i=1_ *p*_i_log(*p*_i_), where *pi* is the proportion of the item *i* (mammal, bird or reptile) on the diet, l*og* is the natural logarithm and *s* is total of diet items. Thus, the lower the Shannon index the less diversified the diet of a given species.

## Supplementary Information

Supplementary Information.

## Data Availability

The data that support the findings of this study are available in the Dryad Digital Repository https://datadryad.org/stash/share/EyCuAmWcmLg55AXfgImAhch4wDovaLrxUXsDW0yr1E8.
