# Supplementary material for: Ecological drivers of breeding periodicity in four forest neotropical eagles
Source: Sci Rep. 2023 Mar 16;13:4385. doi: 10.1038/s41598-023-31274-8 (PMC10020578; doi:10.1038/s41598-023-31274-8)
Supplement: Supplementary file 1 — Supplementary Information. [file 41598_2023_31274_MOESM1_ESM.docx]

**SUPPLEMENTARY FIGURE LEGENGDS**

**Supplementary Fig. 1 –** Map showing the location of the *in situ* and *ex situ* individuals surveyed in this study. Maps were obtained from Abobe Stock (image #289288942) and plots of animal locations were made using Microsoft PowerPoint for Mac Version 16.16.27.

**Supplementary Fig. 2 –** Violin plots of the relative changes in (A) photoperiod, (B) temperature and (C) precipitation in which clutch initiation occurred in captivity for the four Neotropical species surveyed in this study. Red, blue, green and black violin plots represent values obtained from Harpy eagles, Crested eagles, Ornate hawk-eagles and Black hawk-eagles, respectively. Black horizontal lines indicate medians whereas red dots depict means. Boxes illustrate interquartile range and outliers 1.5 times the interquartile range from the box are shown as dots. Different letters indicate statistical difference between species within the same environmental factors (P < 0.05).

**Supplementary Fig. 3 –** Violin plots showing data from captive and free-ranging data regarding relative changes in (A) precipitation and (B) photoperiod when egg laying occurred in Black hawk-eagles and Ornate hawk-eagles, respectively. Black horizontal lines indicate medians whereas red dots depict means. Boxes illustrate interquartile range and outliers 1.5 times the interquartile range from the box are shown as dots. Asterisks indicate statistical differences between captive and wild (P < 0.05).

**Supplementary Fig. 4 -** Principal Component Analysis (PCA) of the variables relative changes in photoperiod, temperature, and precipitation using only the data from captive individuals. The first principal component (x axes) explained 62.5% of the data variation and the second principal component explained 27% of the variation of the data variation. The ellipses define the region that contains 68% of the data for each eagle. The red arrows represent the loadings of each variable (relative photoperiod, relative temperature and relative precipitation). The values of the data in terms of the principal components were multiplied by -1 to facilitate the visualization.

**Supplementary Fig. 5 -** Phylogeny with all the species that share the most recent common ancestor (root of phylogeny) of Harpy eagle (*Harpia harpyja*), Crested eagle (*Morphnus guianensis*), Ornate hawk-eagle (*Spizaetus ornatus*), and Black hawk-eagle (*Spizaetus tyrannus*) (all pointed with red arrows).

SUPPLEMENTARY FIGURE 1


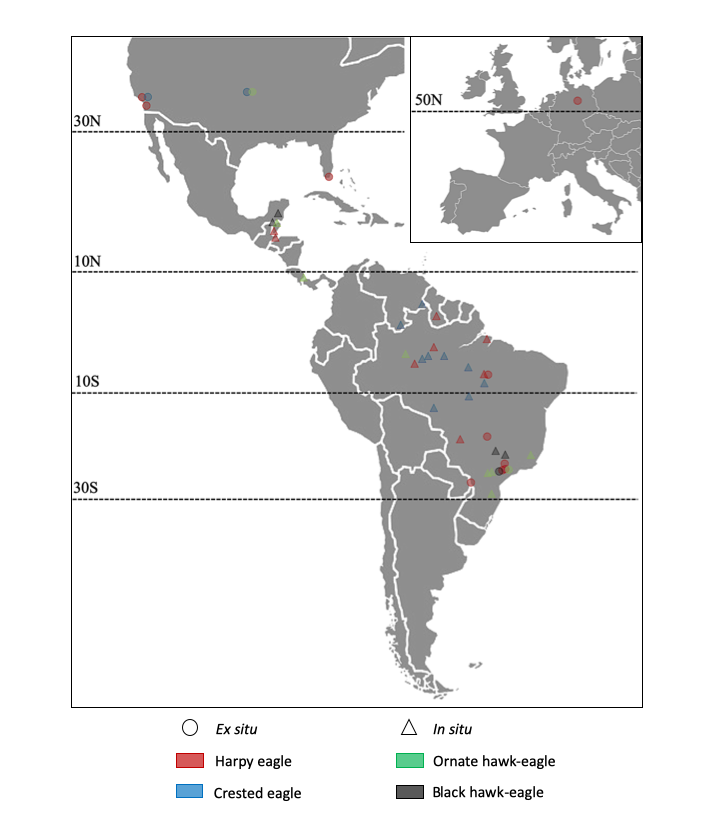


SUPPLEMENTARY FIGURE 2


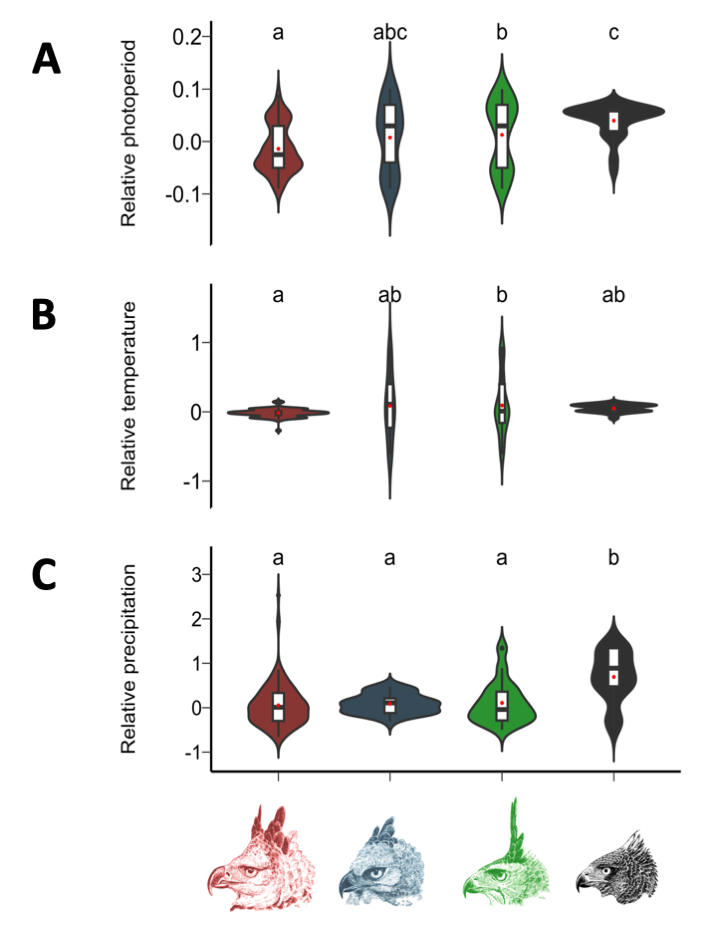


SUPPLEMENTARY FIGURE 3


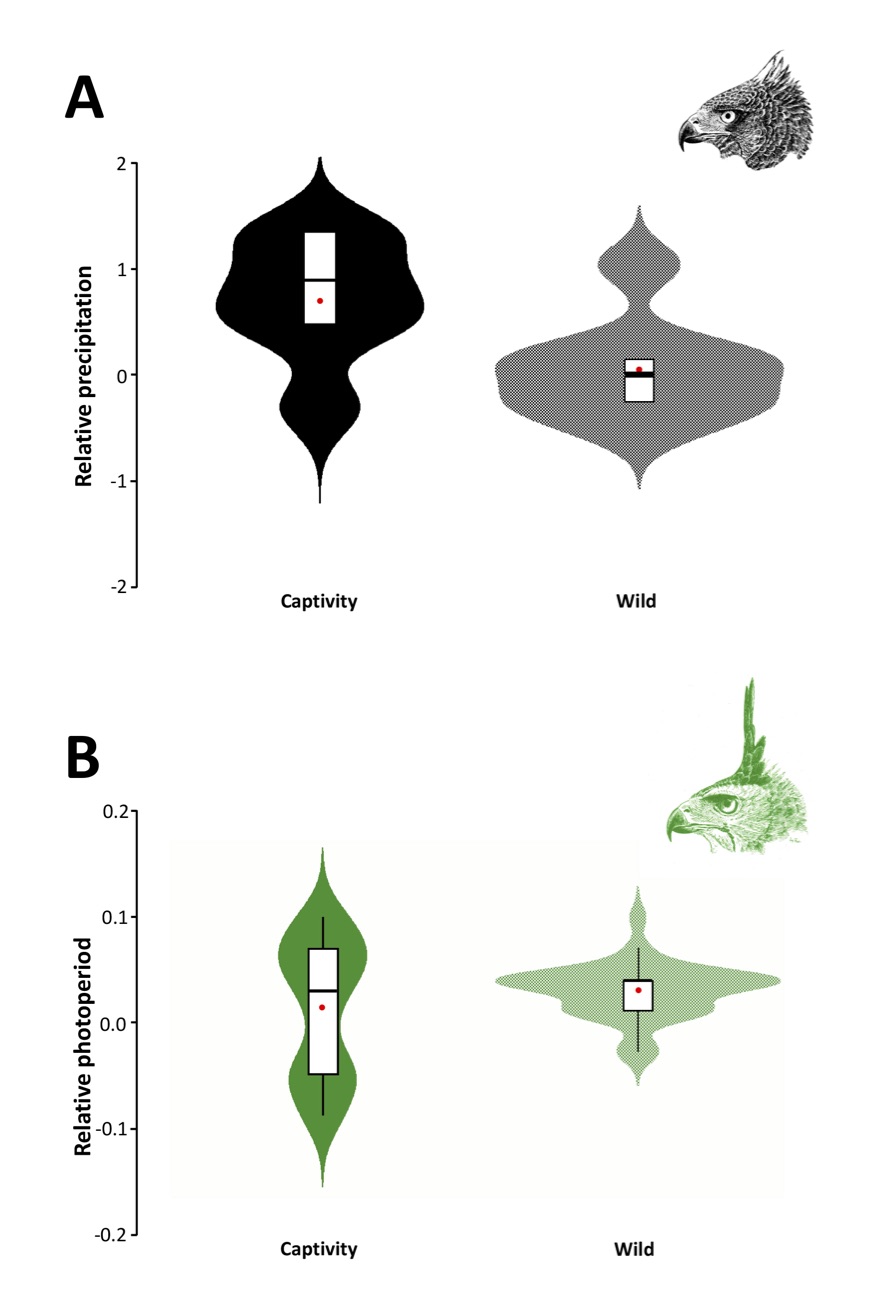


SUPLEMENTARY FIGURE 4


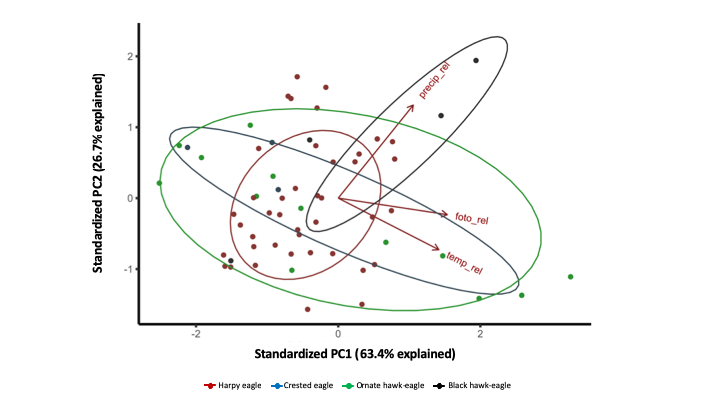


SUPPLEMENTARY FIGURE 5


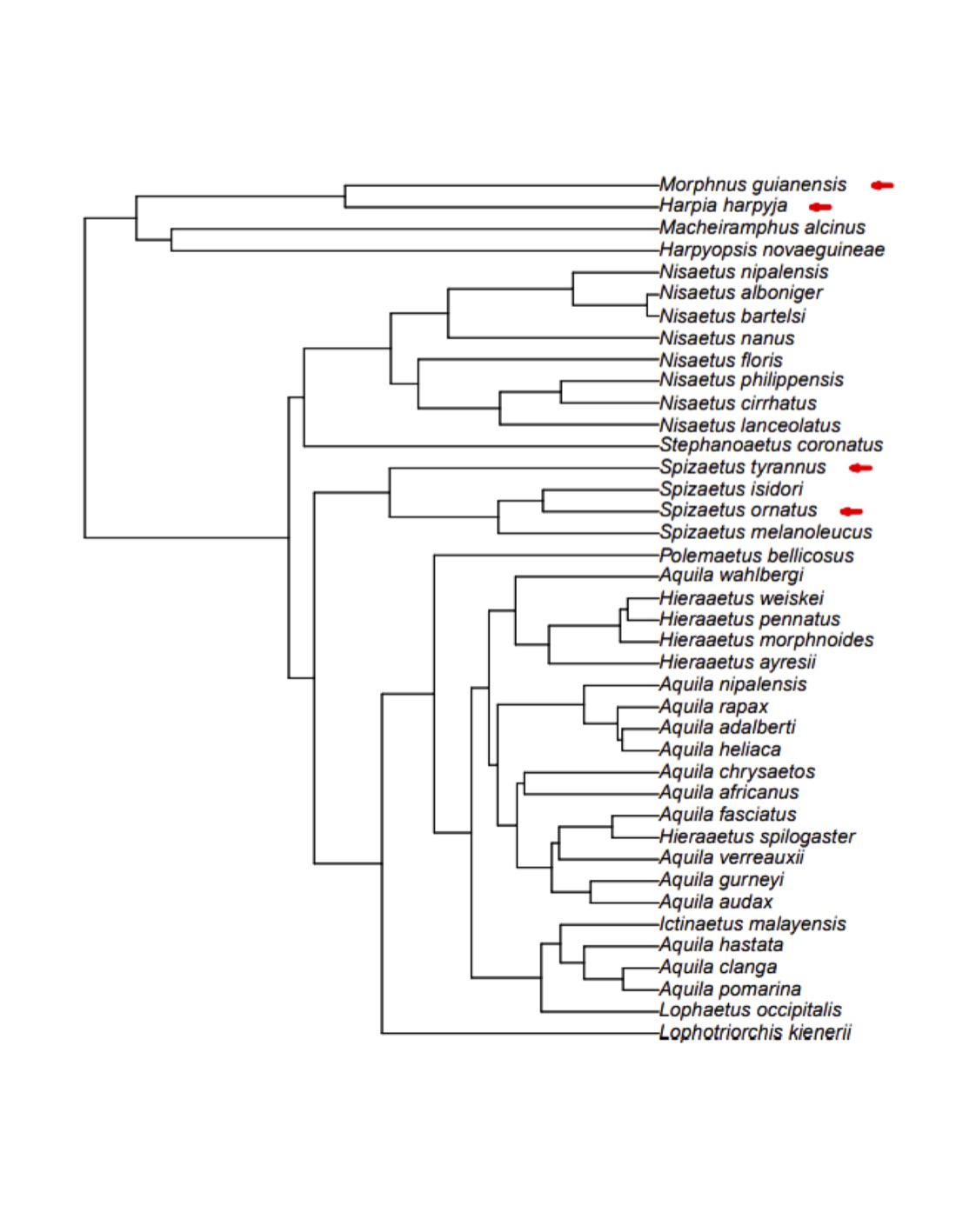


**SUPPLEMENTARY TABLES**

**Supplementary Table S1.** Number of captive and free-ranging pairs surveyed during our study

| **Species** | **Number of captive pairs** | **Number of free-ranging pairs** |
| --- | --- | --- |
| *Ornate hawk-eagle*  *(Spizaetus ornatus)* | 4 | 20 |
| *Black hawk-eagle*  *(Spizaetus tyrannus)* | 1 | 6 |
| *Crested Eagle*  *(Morphnus guianensis)* | 2 | 8 |
| *Harpy eagle*  *(Harpia harpyja)* | 13 | 8 |

**Supplementary Table S2.** Origin of the data collected for each species

| **Species** | **From scientific material*** | **From records in WikiAves** | **From questionnaires** |
| --- | --- | --- | --- |
| *Ornate hawk-eagle*  *(Spizaetus ornatus)* | 8 | 9 | 2 |
| *Black hawk-eagle*  *(Spizaetus tyrannus)* | 3 | 3 | 1 |
| *Crested Eagle*  *(Morphnus guianensis)* | 5 | 4 | 1 |
| *Harpy eagle*  *(Harpia harpyja)* | 7 | 3 | 7 |

**Some peer-reviewed publications, book chapters and thesis documented laying events from more than one pair*

**Supplementary Table S3.** Tukey’s pairwise comparisons among species for relative photoperiod, temperature and precipitation in which egg laying occurred (considering captive and free-ranging data).

| **Species 1** | **Species 2** | **Relative photoperiod** | |  | **Relative temperature** | |  | **Relative precipitation** | |  |
| --- | --- | --- | --- | --- | --- | --- | --- | --- | --- | --- |
|  |  | **Estimate** | **p-value** |  | **Estimate** | **p-value** |  | **Estimate** | **p-value** |  |
| *Harpy eagle* | *Crested eagle* | 0.018 | 0.125 |  | 0.070 | 0.373 |  | 0.028 | 0.983 |  |
| *Harpy eagle* | *Ornate hawk-eagle* | 0.030 | < 0.001 |  | 0.094 | 0.009 |  | 0.053 | 0.731 |  |
| *Harpy eagle* | *Black hawk-eagle* | 0.050 | < 0.001 |  | 0.063 | 0.457 |  | 0.542 | < 0.001 |  |
| *Crested eagle* | *Ornate hawk-eagle* | 0.012 | 0.434 |  | 0.024 | 0.947 |  | 0.025 | 0.987 |  |
| *Crested eagle* | *Black hawk-eagle* | 0.032 | 0.006 |  | - 0.007 | 0.999 |  | 0.513 | < 0.001 |  |
| *Ornate hawk-eagle* | *Black hawk-eagle* | 0.020 | 0.052 |  | - 0.031 | 0.881 |  | 0.488 | < 0.001 |  |

**Supplementary Table S4.** Tukey’s pairwise comparisons among species for relative photoperiod, temperature and precipitation in which egg laying occurred (considering only in captive data)

| **Species 1** | **Species 2** | **Relative photoperiod** | |  | **Relative temperature** | |  | **Relative precipitation** | |  |
| --- | --- | --- | --- | --- | --- | --- | --- | --- | --- | --- |
|  |  | **Estimate** | **p-value** |  | **Estimate** | **p-value** |  | **Estimate** | **p-value** |  |
| *Harpy eagle* | *Crested eagle* | 0.021 | 0.191 |  | 0.105 | 0.287 |  | 0.036 | 0.980 |  |
| *Harpy eagle* | *Ornate hawk-eagle* | 0.026 | <0.001 |  | 0.109 | 0.012 |  | 0.055 | 0.757 |  |
| *Harpy eagle* | *Black hawk-eagle* | 0.054 | <0.001 |  | 0.068 | 0.533 |  | 0.640 | <0.001 |  |
| *Crested eagle* | *Ornate hawk-eagle* | 0.005 | 0.963 |  | 0.004 | 0.999 |  | 0.019 | 0.997 |  |
| *Crested eagle* | *Black hawk-eagle* | 0.032 | 0.057 |  | -0.036 | 0.952 |  | 0.603 | <0.001 |  |
| *Ornate hawk-eagle* | *Black hawk-eagle* | 0.027 | 0.022 |  | -0.041 | 0.852 |  | 0.584 | <0.001 |  |

**Supplementary Table S5.** Contribution of each variable (loadings) to the principal components (considering captive and free-ranging data).

| **Variable** | **PC1** | **PC2** | **PC3** |
| --- | --- | --- | --- |
| Relative photoperiod | 0.657 | -0.151 | -0.737 |
| Relative temperature | 0.606 | -0.474 | 0.637 |
| Relative precipitation | 0.446 | 0.867 | 0.219 |

**Supplementary Table S6.** Contribution of each variable (loadings) to the principal components (considering only captive).

| **Variable** | **PC1** | **PC2** | **PC3** |
| --- | --- | --- | --- |
| Relative photoperiod | 0.655 | -0.153 | -0.739 |
| Relative temperature | 0.604 | -0.478 | 0.636 |
| Relative precipitation | 0.451 | 0.864 | 0.221 |

**Supplementary Table S7:** Phylogenetic distance (in millions of years) between species.

| **Species** | ***Ornate hawk-eagle***  *(Spizaetus ornatus)* | ***Black hawk-eagle***  *(Spizaetus tyrannus)* | ***Harpy eagle***  *(Harpia harpyja)* |
| --- | --- | --- | --- |
| ***Black hawk-eagle***  *(Spizaetus tyrannus)* | 30.639 | ⎯ | ⎯ |
| ***Crested eagle***  *(Morphnus guianensis)* | 65.319 | 65.319 | 35.699 |
| ***Harpy eagle***  *(Harpia harpyja)* | 65.319 | 65.319 | ⎯ |
